# Intrathecal treatment of neoplastic meningitis due to breast cancer with a slow-release formulation of cytarabine

**DOI:** 10.1054/bjoc.2000.1574

**Published:** 2001-01

**Authors:** K A Jaeckle, S Phuphanich, M J van den Bent, R Aiken, T Batchelor, T Campbell, D Fulton, M Gilbert, D Heros, L Rogers, S J O'Day, W Akerley, J Allen, S Baidas, S Z Gertler, H S Greenberg, S LaFollette, G Lesser, W Mason, L Recht, E Wong, M C Chamberlain, A Cohn, M J Glantz, J C Gutheil, B Maria, P Moots, P New, C Russell, W Shapiro, L Swinnen, S B Howell

**Affiliations:** From the M.D. Anderson Cancer Center; H. Lee Moffitt Cancer Center; Dr. Daniel den Hoed Cancer Clinic; Thomas Jefferson University Hospital; Massachusetts General Hospital; Sharp Healthcare; Cross Cancer Institute; the Emory Clinic; Mt. Sinaiomprehensive Cancer Center; Wayne State University Health Center; the John Wayne Cancer Institute; Memorial Hospital of Rhode Island; New York University Medical Center; Georgetown University Medical Center; Ottowa Regional Cancer Center; University ofichigan Hospitals; Rush Cancer Institute; Comprehensive Cancer Center of Wake Forrest University; Ontario Cancer Center; University of Massachusetts Medical School; Beth Israel Deaconess Medical Center; University of California San Diego; University of colorado; University of Florida; Vanderbilt University Medical Center; University of Texas, San Antonio; Norris Cancer Center; Barrow Neurological Institute; and Loyola University

**Keywords:** breast cancer, neoplastic meningitis, cytarabine, drug delivery

## Abstract

DepoCyte is a slow-release formulation of cytarabine designed for intrathecal administration. The goal of this multi-centre cohort study was to determine the safety and efficacy of DepoCyte for the intrathecal treatment of neoplastic meningitis due to breast cancer. DepoCyte 50 mg was injected once every 2 weeks for one month of induction therapy; responding patients were treated with an additional 3 months of consolidation therapy. All patients had metastatic breast cancer and a positive CSF cytology or neurologic findings characteristic of neoplastic meningitis. The median number of DepoCyte doses was 3, and 85% of patients completed the planned 1 month induction. Median follow up is currently 19 months. The primary endpoint was response, defined as conversion of the CSF cytology from positive to negative at all sites known to be positive, and the absence of neurologic progression at the time the cytologic conversion was documented. The response rate among the 43 evaluable patients was 28% (CI 95%: 14–41%); the intent-to-treat response rate was 21% (CI 95%: 12–34%). Median time to neurologic progression was 49 days (range 1–515^+^); median survival was 88 days (range 1–515^+^), and 1 year survival is projected to be 19%. The major adverse events were headache and arachnoiditis. When drug-related, these were largely of low grade, transient and reversible. Headache occurred on 11% of cycles; 90% were grade 1 or 2. Arachnoiditis occurred on 19% of cycles; 88% were grade 1 or 2. DepoCyte demonstrated activity in neoplastic meningitis due to breast cancer that is comparable to results reported with conventional intrathecal agents. However, this activity was achieved with one fourth as many intrathecal injections as typically required in conventional therapy. The every 2 week dose schedule is a major advantage for both patients and physicians. © 2001 Cancer Research Campaign http://www.bjcancer.com


*British Journal of Cancer* (2001) **84**(2), 157–163
© 2001 Cancer Research Campaign
doi: 10.1054/ bjoc.2000.1574, available online at http://www.idealibrary.com on IDEAL®

### Intrathecal treatment of neoplastic meningitis due to breast cancer with a slow-release formulation of cytarabine

**KA Jaeckle, S Phuphanich, MJ van den Bent, R Aiken, T Batchelor, T Campbell, D Fulton, M Gilbert, D Heros, L Rogers, SJ O'Day, W Akerley, J Allen, S Baidas, SZ Gertler, HS Greenberg, S LaFollette, G Lesser, W Mason, L Recht, E Wong, MC Chamberlain, A Cohn, MJ Glantz, JC Gutheil, B Maria, P Moots, P New, C Russell, W Shapiro, L Swinnen and SB Howell**

From the M.D. Anderson Cancer Center; H. Lee Moffitt Cancer Center; Dr. Daniel den Hoed Cancer Clinic; Thomas Jefferson University Hospital; Massachusetts General Hospital; Sharp Healthcare; Cross Cancer Institute; the Emory Clinic; Mt. Sinai Comprehensive Cancer Center; Wayne State University Health Center; the John Wayne Cancer Institute; Memorial Hospital of Rhode Island; New York University Medical Center; Georgetown University Medical Center; Ottowa Regional Cancer Center; University of Michigan Hospitals; Rush Cancer Institute; Comprehensive Cancer Center of Wake Forrest University; Ontario Cancer Center; University of Massachusetts Medical School; Beth Israel Deaconess Medical Center; University of California, San Diego; University of Colorado; University of Florida; Vanderbilt University Medical Center; University of Texas, San Antonio; Norris Cancer Center; Barrow Neurological Institute; and Loyola University

**Summary** DepoCyte is a slow-release formulation of cytarabine designed for intrathecal administration. The goal of this multi-centre cohort study was to determine the safety and efficacy of DepoCyte for the intrathecal treatment of neoplastic meningitis due to breast cancer. DepoCyte 50 mg was injected once every 2 weeks for one month of induction therapy; responding patients were treated with an additional 3 months of consolidation therapy. All patients had metastatic breast cancer and a positive CSF cytology or neurologic findings characteristic of neoplastic meningitis. The median number of DepoCyte doses was 3, and 85% of patients completed the planned 1 month induction. Median follow up is currently 19 months. The primary endpoint was response, defined as conversion of the CSF cytology from positive to negative at all sites known to be positive, and the absence of neurologic progression at the time the cytologic conversion was documented. The response rate among the 43 evaluable patients was 28% (CI 95%: 14–41%); the intent-to-treat response rate was 21% (CI 95%: 12–34%). Median time to neurologic progression was 49 days (range 1–515$^+$); median survival was 88 days (range 1–515$^+$), and 1 year survival is projected to be 19%. The major adverse events were headache and arachnoiditis. When drug-related, these were largely of low grade, transient and reversible. Headache occurred on 11% of cycles; 90% were grade 1 or 2. Arachnoiditis occurred on 19% of cycles; 88% were grade 1 or 2. DepoCyte demonstrated activity in neoplastic meningitis due to breast cancer that is comparable to results reported with conventional intrathecal agents. However, this activity was achieved with one fourth as many intrathecal injections as typically required in conventional therapy. The every 2 week dose schedule is a major advantage for both patients and physicians. © 2001 Cancer Research Campaign http://www.bjcancer.com

**Keywords:** breast cancer; neoplastic meningitis; cytarabine; drug delivery

Neoplastic meningitis complicating metastatic breast cancer often portends a progressive rapid neurologic deterioration to a fatal outcome. Neoplastic meningitis most commonly occurs in patients with evidence of metastases at other sites. Effective treatment usually requires control of both the meningeal and systemic components of the disease. Treatment of the meningeal disease is problematic because so few anticancer drugs attain therapeutic concentrations in the CSF when administered by the intravenous route. The standard therapeutic approach includes limited-field radiation therapy to symptomatic sites or radiographically definable areas of bulk disease, and administration of methothrexate in doses of 5 to 12 mg intrathecally 2–3 times a week, usually in combination with oral leucovorin (Howell, 1997). However, while methotrexate is active against this disease, the CSF half-life is only 4.5–8 h (Bleyer and Dedrick, 1977; Shapiro et al, 1975), so twice a week dosing is not a pharmacokinetically optimal schedule. High doses of methotrexate administered systemically can maintain effective CSF concentrations for somewhat longer periods of time and clear this compartment of malignant cells in a large fraction of cases (Glantz et al, 1998), but in many patients it is difficult to integrate this regimen into other chemotherapeutic programmes being used to control systemic disease. Not surprisingly, therapeutic outcome is generally poor and there are very few long term survivors (Ongerboer de Visser et al, 1983; Giannone et al, 1986; Hitchins et al, 1987; Nakagawa et al, 1992; Grossman et al, 1993; Grant et al, 1994).

Cytarabine (ara-C) is a cell cycle phase-specific agent whose cytotoxicity is a function of both drug concentration and duration of exposure. Maximum tumour cell kill is attained by maintaining high concentrations in the micro-environment of the cancer cell for prolonged periods of time (Graham and Whitmore 1970). DepoCyte is a slow-release formulation of ara-C developed

*Received 3 May 2000*
*Revised 8 September 2000*
*Accepted 11 October 2000*

*Correspondence to:* Stephen B. Howell, M.D., Department of Medicine 0058, University of California, San Diego, La Jolla, CA 92093

specifically for the treatment of neoplastic meningitis (Kim et al, 1993). In DepoCyte the ara-C is encapsulated in the aqueous chambers of a spherical 20 um matrix comprised of lipids biochemically similar to normal human cell membranes (phospholipids, triglycerides and cholesterol). The particles are suspended in 0.9% NaCl saline. When stored at 2–8˚C, the drug remains inside the particle; when injected into the CSF the particles spread out throughout the neuraxis and, at the ambient temperature of 37˚C, slowly release the ara-C. The particles eventually disappear from CSF and the lipids undergo degradation (Kohn et al, 1998). Whereas a single injection of free unencapsulated ara-C maintains cytotoxic concentrations in the CSF for <24 hours (Zimm et al, 1984), a single injection of 50 mg of DepoCyte maintains cytotoxic concentrations of ara-C in the CSF for >14 days in most patients, and the drug distribution within the neuraxis is relatively homogeneous (Kim et al, 1993). Thus, intrathecal administration of DepoCyte is required just once every 2 weeks as compared to a daily dose schedule which would theoretically be required to maintain cytotoxic concentrations in the CSF following injection of free unencapsulated ara-C.

This paper reports on the efficacy and safety of DepoCyte administered to the 51 patients with neoplastic meningitis complicating their breast cancer.

### MATERIALS AND METHODS

### Study design

The breast cancer patients included in this report were drawn from 4 different trials which were open to patients with neoplastic meningitis due to any type of solid tumour. The first was an open label, randomized, parallel-group multicentre trial in which patients with cytologically documented solid tumour neoplastic meningitis were treated with either intrathecal DepoCyte or with intrathecal methotrexate (Glantz et al, 1999). 11 of the patients included in the current report were treated in this previously reported study. The other 3 trials were open-label, non-randomized single arm trials the results of which have not been published. All trials utilized the same eligibility criteria, dose schedule, definition of response, and safety evaluation criteria. After informed consent was obtained, patients were entered from a total of 21 institutions with the approval of appropriate Institutional Review Boards; accrual commenced in July, 1994 and the last patient was entered in April 1999. The data cutoff date for this analysis was 8 August 2000.

### Patients

The inclusion criteria required that patients have histologically proven breast cancer, a Karnofsky Performance Status > 50%, no uncontrolled infection other than HIV, and recovery from toxicities of prior intrathecal treatment. All but 3 patients had a positive CSF cytology within 14 days prior to entry; these 3 patients were diagnosed on the basis of characteristic neurologic and MRI scan findings. Required laboratory values for treatment were platelets ≥ 80 000 and WBC≥ 3000/mm$^3$ (or ANC ≥ 1000/mm$^3$), creatinine ≤2 times the upper limit of normal and all other blood chemistries < 3 times normal. Prior CNS radiation of any type was allowed if completed any time up to the day of study entry, but prior IT methotrexate for the treatment of known neoplastic meningitis was not. Patients entering the study with symptomatic or radiologically visible CNS disease were required to receive local radiation therapy during the induction period but concurrent whole brain or craniospinal radiation was not permitted. CSF compartmentalization identified on a radioisotope flow study, or the need for a ventriculo-peritoneal shunt excluded the patient from participation. Concurrent systemic chemotherapy was permitted for treatment of disease outside of the meninges, but high dose methotrexate (> 500 mg/m$^2$ day$^{-1}$), high dose ara-C (> 2 g/m$^2$ day$^{-1}$), high dose thio TEPA (> 300 mg/m$^2$ day$^{-1}$) or investigational agents were not allowed.

### DepoCyte treatment

Figure 1 presents a schematic diagram of the study design. Patients received DepoCyte 50 mg by intraventricular (IVT) or lumbar sac (LP) injection once every 14 days for 2 doses during the induction phase. Those responding at the end of the induction phase then received 50 mg once every 14 days for 1 month and then once a month for 2 months for a total of 3 months of consolidation therapy. Unless already receiving dexamethasone, all patients were treated with dexamethasone 4 mg bid p.o. or i.v. on days 1–5 of each cycle beginning concurrently with administration of the DepoCyte.

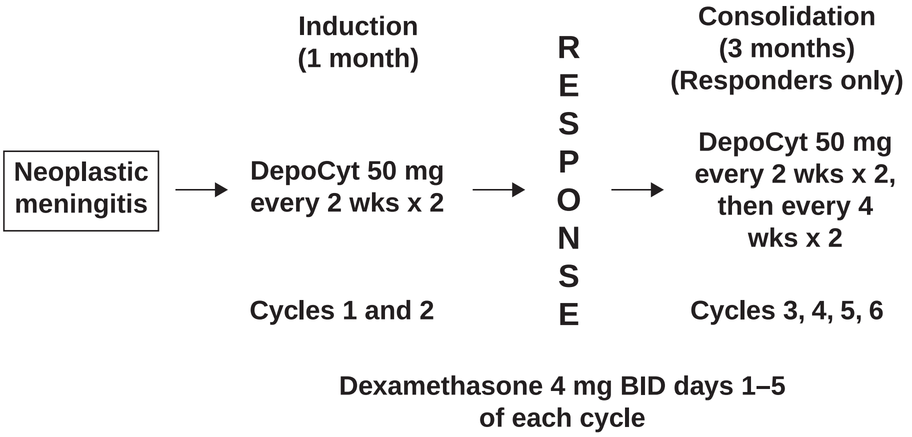

**Figure 1** Study schema

 *© 2001 Cancer Research Campaign*

**Table 1** Baseline characteristics of possible prognostic significance

| | |
|---|---|
| Total number of patients | 56 |
| Median age, yrs (range) | 50 (28–74) |
| Race | |
| White | 44 (78%) |
| Non-white | 12 (21%) |
| Median Karnofsky score | 80 |
| Prior IT chemotherapy | 3[a] (5%) |
| Prior CNS irradiation | 13 (25%) |
| Concurrent systemic chemotherapy | 12 (22%) |
| Concurrent CNS irradiation | 13 (23%) |

[a]Single dose of IT methotrexate only

### Clinical and laboratory monitoring

Before each cycle of therapy, patients underwent a complete neurologic history and exam, measurement of haematologic and serum chemistry parameters, a urinalysis, and assessment of CSF cytology. An independent cytopathologist, blinded to the chronology of CSF samples, reviewed all available CSF cytology slides after the patient completed the study. All treatment decisions (whether or not to continue therapy) were based on the reading of the local cytopathologist. Efficacy analyses were based on central cytology review except when the slide could not be recovered. In these cases, the report of the local institutional cytopathologist was utilized.

Adverse events were graded according to the NCI common Toxicity Criteria. Identification of episodes of arachnoiditis was based on a standardized algorithm. Patients were scored as having drug-related arachnoiditis if, within 4 days of drug injection, they developed either neck rigidity, neck pain, or meningismus, or if they developed any 2 of the following signs or symptoms at the same time: nausea, vomiting, headache, fever, back pain, or aseptic CSF pleocytosis. Arachnoiditis was graded on the basis of the highest grade of any of the constellation of adverse events captured by the algorithm as mild (grade 1), moderate (grade 2), severe (grade 3), or life-threatening (grade 4).

### Assessment of response

Patients were only scored as responders if their CSF cytology converted from positive to negative at all sites (i.e. ventricle and/or lumbar sac) previously shown to be positive, and they remained neurologically stable by objective criteria at the time of the CSF conversion. Patients were scored as non-responders if they had a positive or suspicious cytology at the end of the induction period (day 29), or if they suffered neurologic progression regardless of CSF cytology status. Patients were considered evaluable for response if they had a positive CSF cytology prior to treatment, completed at least one cycle of DepoCyte therapy, and had adequate post-treatment CSF cytologic analysis. Time to neurologic progression was defined as the time between the first day of study treatment and the day the patient was scored as suffering neurologic progression on the basis of the physician's global assessment of neurologic status, or until death, whichever came first.

### RESULTS

A total of 56 women with breast cancer were registered for treatment. Figure 1 presents a schematic diagram of the treatment plan. 53 patients actually received DepoCyte; 3 patients refused treatment after initially consenting to study participation. 8 patients received treatment exclusively via lumbar puncture, 42 patients received intraventricular treatment exclusively via an Ommaya reservoir, and 3 patients received at least one injection by both routes. Table 1 shows the baseline demographic characteristics of previously reported prognostic significance in solid tumour neoplastic meningitis (Hitchins et al, 1987; Grossman et al, 1993), including age, gender, race, presence or absence of AIDS and Karnofsky performance status.

As shown in Figure 2, a total of 177 treatment cycles were given to the 53 patients who received drug (median 3 cycles/patient). 45 of the 53 (85%) patients receiving drug were able to complete the planned 1 month of induction therapy; 8 (15%) were able to complete planned 3 month consolidation phase of treatment. The median time on study was 87 days (range 1–797 days), and the median follow up was 571 days with a range of 74–1313 days.

10 of the 53 patients who received DepoCyt were not evaluable for response; 3 did not have a positive baseline cytology on central review, and 7 did not have sufficient post-treatment CSF cytology examinations to permit assessment. 12 of the 43 patients evaluable for response (28%) attained a response (95% confidence interval 14 to 41%). On an intent-to-treat basis the response rate was 21% (95% confidence interval 12 to 34%). There was no association between response and prior intrathecal chemotherapy, concurrent systemic chemotherapy, or prior or concurrent CNS irradiation. For example, whereas 3 of the 12 (25%) patients receiving concurrent systemic chemotherapy attained a response, 9 of the 44 (20%) patients not receiving concurrent systemic chemotherapy responded.

Figure 3 shows a Kaplan-Meier plot of time to neurologic progression for all patients registered. The median was 49 days (range 1 to $515^+$ days); 11% of patients were projected to remain free of neurologic progression at 6 months. Figure 4 shows a Kaplan-Meier plot of overall survival. Median survival was 88 days (range 1 to $515^+$ days), and projected survival at 1 year was 9%.

Data on change in Karnofsky score between baseline and the end of the induction were available for 42 patients. The median pre-treatment Karnofsky score was 80. Table 2 shows that 2% of the patients had an improved score, 43% had no change, and 55% had a worse score at the end of the induction phase of treatment. The mean change in score in the 42 patients actually completing the 1 month induction phase of treatment was –10± 14% (SD).

Table 3 presents information on adverse events. The drug-related adverse events were generally transient, and resolved by the end of the treatment cycle on which they occurred. The only adverse events to occur on ≥10% of cycles were headache, nausea, vomiting and arachnoiditis. Headache occurred on 11% of cycles, but of all the headaches that occurred, 90% were grade 1 or 2. It was difficult to distinguish arachnoiditis caused by meningeal tumour infiltration from that caused by

**Table 2** Change in Karnofsky performance score during the induction phase (to day 29)

| | |
|---|---|
| No. patients with data available | 42 |
| Improved score | 1 (2%) |
| No change | 18 (43%) |
| Worse score | 23 (55%) |
| Mean change in score[a] | –10 ± 14% |

[a]End of Induction – Baseline, mean ± SD

© 2001 Cancer Research Campaign

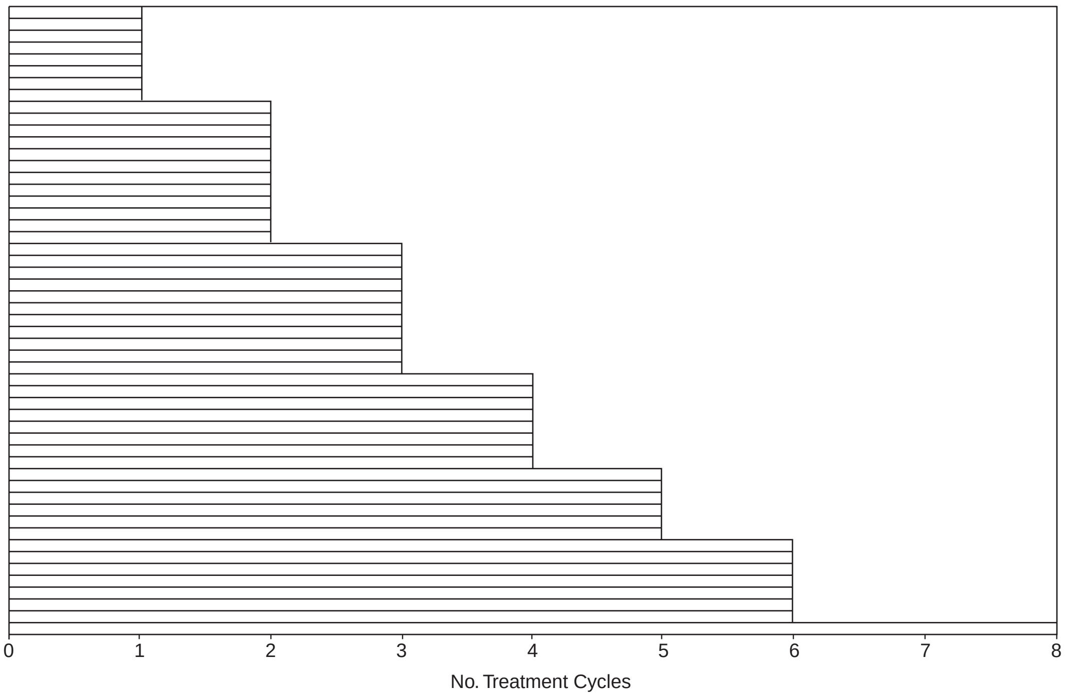

**Figure 2** Distribution of number of cycles of treatment received. Each horizontal bar represents an individual patient; the length of the bar indicates the number of cycles received. Planned treatment consisted of 2 cycles during the induction phase, and 4 during the consolidation phase

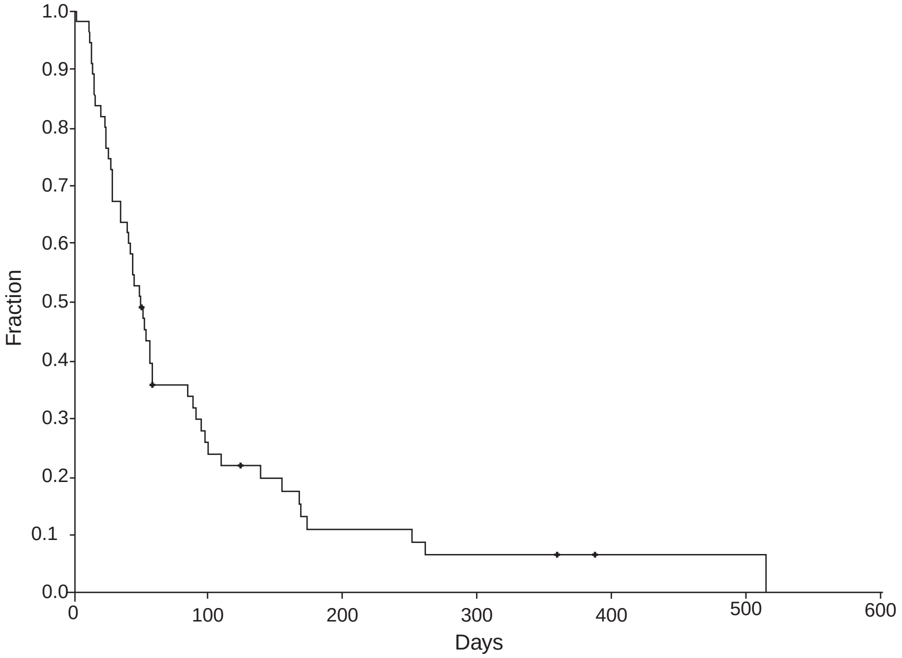

**Figure 3** Kaplan-Meier plots of time to neurologic progression. Hash marks indicate patients who had not yet suffered neurologic progression as of the data cutoff date intrathecal drug administration. As shown in Table 3, arachnoiditis occurred on 19% of cycles; of all the episodes of arachnoiditis, 88% were grade 1 or 2. Only 3 patients discontinued DepoCyte treatment in association with an adverse event of any kind (drug-related or non-drug-related). There was no evidence of any form of cumulative toxicity due to DepoCyte treatment.

### DISCUSSION

This series constitutes the largest group of breast cancer patients with neoplastic meningitis treated with intrathecal agents reported to date. All patients were prospectively treated according to the same eligibility criteria, and with the same intrathecal chemotherapy

 *© 2001 Cancer Research Campaign*

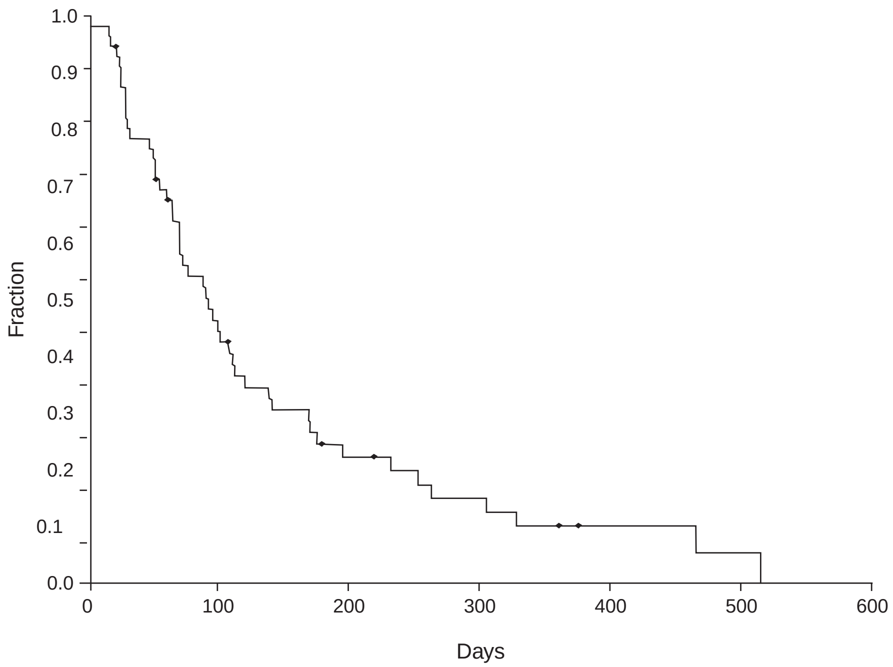

**Figure 4** Kaplan-Meier plots of survival. Hash marks indicate patients alive at the data cutoff date regimen. Strict response and adverse event monitoring procedures were utilized with blinded central cytology review. The patients included in these trials were comparable to those reported in prior series with regard to variables previously reported to be of prognostic significance in this disease (Boogerd et al, 1991; Grossman et al, 1993; Jayson et al, 1994; Balm and Hammack, 1996; Fizazi et al, 1996; Chamberlain et al, 1997).

In patients with neoplastic meningitis, spontaneous clearing of malignant cells from the CSF in combination with lack of neurologic progression is uncommon. Thus, the response rate of 28%, with a 95% confidence interval of from 14–41%, observed in this trial likely reflects drug effect. It is important to note that the definition of response in these patients was quite stringent, requiring both conversion of the CSF cytology from positive to negative, and absence of neurologic progression according to objective criteria. Methotrexate is the drug that is currently most commonly used for the treatment of solid tumour neoplastic meningitis. Prior to the development of DepoCyte, only two prospective controlled trials were reported in patients with solid tumour neoplastic meningitis. In the first of these (Hitchins et al, 1987), the fraction of patients clearing their CSF of malignant cells was not reported. In the second (Grossman et al, 1993), the definition of response approximated that used in the current trial, and 8 of 28 patients (29%) cleared their CSF of malignant cells. In making comparisons of response rates, it is important to note that the DepoCyte response rate of 28% reported here was attained with dosing once every 2 weeks, one fourth to one sixth of the customary methotrexate dosing frequency. None of the former trials were conducted with the same rigor as the current studies, and none included a central blinded cytology review.

Many clinicians have a nihilistic attitude toward the treatment of solid tumour neoplastic meningitis. In part, this attitude is justified due to poor responses, lack of clinical improvement even when the CSF is cleared of malignant cells, and because patients with neoplastic meningitis often have extensive systemic end stage disease. However, there is a subset of patients in whom the meningeal involvement is either the only known site of disease, or the site that most directly threatens quality of life or survival. In this subset, intrathecal treatment may be warranted as adequate control of the meningeal disease may offer substantial benefit in

**Table 3** Number (%) of cycles on which a drug-related adverse event of the indicated grade occurred

| | | | No. cycles = 177 | | |
|---|---|---|---|---|---|
| **Grade of toxicity** | **1** | **2** | **3** | **4** | **All Grades** |
| Headache | 6[a] (3%)[b] | 12 (7%) | 1 (0.5%) | 1 (0.5%) | 20 (11%) |
| Nausea | 8 (5%) | 12 (7%) | 1 (0.5%) | 1 (0.5%) | 22 (12%) |
| Vomiting | 7 (4%) | 11 (6%) | 0 | 0 | 18 (10%) |
| Arachnoiditis | 17 (10%) | 12 (7%) | 3 (2%) | 1 (0.6%) | 33 (19%) |

[a]Includes all adverse events that were possibly, probably or definitely drug-related, or for which relationship to drug could not be determined which occurred on ≥ 10% of cycles. [b]Number of cycles in which the AE of the specified type and grade occurred.[c]Percent of total cycles on which the AE occurred.

© 2001 Cancer Research Campaign the form of prolonged time to neurologic progression and possibly survival.

The median time to neurologic progression was 49 days for the breast cancer patients included in this group of studies. This is similar to that of 58 days reported for DepoCyte-treated patients with various forms of solid tumour neoplastic meningitis treated in a prospective randomized controlled trial in which this drug was compared to methotrexate (Glantz et al, 1999). In this controlled trial, methotrexate treatment yielded a median time to neurologic progression of 30 days which was significantly inferior to that produced by DepoCyte ($P < 0.007$). Many patients with neoplastic meningitis die of systemic or combined systemic and meningeal disease, and as a result adequate control of the meningeal disease may not impact on survival. The median overall survival of 88 days observed in the breast cancer patients treated with DepoCyte was comparable to that reported in prior studies of this drug (Glantz et al, 1999). Although a number of investigators have reported on the treatment of neoplastic meningitis due to breast cancer (Ongerboer de Visser et al, 1983; Boogerd et al, 1991; Jayson et al, 1994; Fizazi et al, 1996; Chamberlain and Kormanik 1997), in none of these studies did all patients meet the same eligibility criteria, receive the same treatment in a prospective manner, and undergo evaluation on the basis of the same prospectively defined outcome measures. Thus, valid comparisons of time to neurologic progression and survival cannot be made. Nevertheless, the average median survival reported was 13 weeks, leaving open the question of whether DepoCyt improves overall survival in this disease.

Is it reasonable to expect that a slow-release formulation of ara-C would have significant activity against neoplastic meningitis due to breast cancer? Ara-C is a cell cycle specific antimetabolite whose cytotoxicity is very sensitive to duration of exposure. When ara-C was tested in the NCI 60 cell line panel, the median concentration required to inhibit growth by 50% was 17 uM when the exposure duration was 2 days, and 0.17 uM when the cells were exposed for 6 days. Thus, a relatively modest increase in the duration of exposure increased efficacy by 2 orders of magnitude. Intrathecal administration of DepoCyte results in peak free ara-C concentrations of 300 μM that decay with a half-life of 144 hours (Kim et al, 1993). This is in contrast to a CSF half-life of 3.4 hours for free unencapsulated ara-C (Zimm et al, 1984), and a CSF half-life of 4.5–8 hours for methotrexate (Shapiro et al, 1975; Bleyer and Dermick, 1977). Thus, it is reasonable to expect that DepoCyte should produce a response rate at least comparable to that of standard agents even when administered less frequently.

The most common adverse events associated with DepoCyte therapy were those typical of other intrathecally administered chemotherapeutic agents, including headache, nausea, vomiting, and arachnoiditis. The only adverse events that occurred on >10% of cycles were headache, nausea, vomiting and arachnoiditis. It is important to note that a large fraction of patients with neoplastic meningitis due to breast cancer have headache, as well as other symptoms and signs of meningeal irritation due to tumour infiltration of the meninges, prior to the start of treatment. Arachnoiditis occurred on 19% of cycles, and 88% of the episodes were grade 1 or 2. Signs and symptoms of arachnoiditis generally resolved in <4 days. 3 patients discontinued therapy in association with an adverse event, but in none of these cases was cessation of therapy due to the adverse event. The development of an episode of arachnoiditis did not delay any subsequent on-schedule DepoCyte therapy. We conclude that the toxicity of DepoCyte is acceptable in this patient cohort.

The optimal duration of therapy of solid tumour neoplastic meningitis in responding patients has not been defined for any drug. When intrathecal methotrexate is used, it is common practice to dose 2–3 times per week until the CSF is clear of malignant cells, and then at less frequent intervals for weeks to months depending on the status of other components of the disease. This same approach was adopted for the breast cancer patients reported here, although since DepoCyte maintains ara-C concentrations such a prolonged period of time the dosing interval during the initial phase of treatment was 2 weeks, and that during the consolidation phase was 4 weeks. This approach is consistent with the recently issued practice guidelines for the management of neoplastic meningitis (Grossman and Spence, 1999).

This analysis provides response rates, and time to neurologic progression and survival data that can be compared with future trials of intrathecal therapy in breast cancer patients. Further improvements in therapy are urgently needed. Exploration of combination intrathecal chemotherapy with DepoCyte and methotrexate, or systemically administered high-dose methotrexate and intrathecal DepoCyte are warranted.

### ACKNOWLEDGEMENTS

The authors thank G Barry Schulman for reading CSF cytologies, and recognize the contributions of all the patients who participated in these trials, particularly those whose courage in accepting randomization has benefited other women with this disease. Support for this study was provided by SkyePharma, Inc. (San Diego, CA) and the Chiron Corporation (Emeryville, CA).

### REFERENCES

Balm M and Hammack J (1996) Leptomeningeal carcinomatosis – presenting features and prognostic factors. *Arch Neurol* **53**: 626–632

Bleyer WA and Dedrick RL (1977) Clinical pharmacology of intrathecal methotrexate. I. Pharmacokinetics in nontoxic patients after lumbar injection. *Cancer Treat Rep* **61**: 703–708

Boogerd W, Hart AAM, Van der Sande JJ and Engelsman E (1991) Meningeal carcinomatosis in breast cancer. *Cancer* **67**: 1685–1695

Chamberlain M and Kormanik P (1997) Prognostic significance of coexistent bulky metastatic central nerous system disease in patients with leptomeningeal metastases. *Arch Neurol* **54**: 1364–1368

Chamberlain MC and Kormanik PRN (1997) Carcinomatous meningitis secondary to breast cancer: predictors of response to combined modality therapy. *J Neurooncol* **35**: 55–64

Fizazi K, Asselain B, Vincent-Salomon A, Jouve M, Dieras V, Palangie T, Beuzeboc P, Dorval T and Pouillart P (1996) Meningeal carcinomatosis in patients with breast carcinoma. *Cancer* **77**: 1315–1323

Giannone L, Greco FA and Hainsworth JD (1986) Combination intraventricular chemotherapy for meningeal neoplasia. *J Clin Oncol* **4**: 68–73

Glantz MJ, Cole BF, Recht L, Akerley W, Mills P, Saris S, Hochberg F, Calabresi P and Egorin MJ (1998) High-dose intravenous methotrexate for patients with nonleukemic leptomeningeal cancer: Is intrathecal chemotherapy necessary? *J Clin Oncol* **16**: 1561–1567

Glantz M, Jaeckle KA, Chamberlain MC, Phuphanich S, Recht L, Swinnen LJ, Maria B, LaFollette S, Schumann GB, Cole BF and Howell SB (1999) A randomized controlled trial comparing intrathecal sustained-release cytarabine (DepoCyt) to intrathecal methotrexate in patients with neoplastic meningitis from solid tumors. *Clin Cancer Res* **5**: 3394–3402

Graham FL and Whitmore GF (1970) The effect of 1-β-D-arabinofuranosyl-cytosine on growth, viability, and DNA synthesis of mouse L-cells. *Cancer Res* **30**: 2627–2635

Grant R, Naylor B, Greenberg HS and Junck L (1994) Clinical outcome in aggresively treated meningeal carcinomatosis. *Arch Neurol* **51**: 457–461

Grossman SA and Spence A (1999) NCCN clinical practice guidelines for carcinomatous/lymphomatous meningitis. *Oncol* **13**: 144–152

 *© 2001 Cancer Research Campaign*

Grossman SA, Finkelstein DM, Ruckdeschel JC, Trump DL, Moynihan T and Ettinger DS (1993) Randomized prospective comparison of intraventricular methotrexate and thiotepa in patients with previously untreated neoplastic meningitis. *J Clin Oncol* **11**: 561–569

Hitchins RN, Bell DR, Woods RL and Levi JA (1987) A prospective randomized trial of single-agent versus combination chemotherapy in meningeal carcinomatosis. *J Clin Oncol* **5**: 1655–1662

Howell SB (1997) Regional chemotherapy. In: Holland J, Bast RC, Jr. Morton DL, Frei E. III, Kufe DW and Weichselbau RR (eds.) *Cancer Medicine,* Fourth edition. Lea and Febiger: Philadelphia, pp 839–853

Jayson GC, Howell A, Harris M, Morgenstern G, Chang J and Ryder WD (1994) Carcinomatous meningitis in patients with breast cancer. An aggressive disease variant. *Cancer* **74**: 3135–3141

Kim S, Chatelut E, Kim JC, Howell SB, Cates C, Kormanik PA and Chamberlain MC (1993) Extended CSF cytarabine exposure following intrathecal administration of DTC 101. *J Clin Oncol* **11**: 2186–2193

Kohn FR, Malkmus SA, Brownson EA, Rossi SS and Yaksh TL (1998) Fate of the predominant phospholipid component of DepoFoam drug delivery matrix after intrathecal administration of sustained-release encapsulated cytarabine in rats. *Drug Delivery* **5**: 143–151

Nakagawa H, Murasawa A, Kubo S, Nakajima S, Nakajima Y, Izumoto S and Hayakawa T (1992) Diagnosis and treatment of patients with meningeal carcinomatosis. *J Neurooncol* **13**: 81–89

Ongerboer de Visser BW, Somers R, Nooyen WH, van Heerde P, Hart AAM and McVie JG (1983) Intraventricular methotrexate therapy of leptomeningeal metastasis from breast carcinoma. *Neurol* **33**: 1565–1572

Shapiro WR, Young DF and Mehta BM (1975) Methotrexate distribution in cerebrospinal fluid after intravenous, ventricular and lumbar injections. *New Engl J Med* **293**: 161–166

Zimm S, Collins JM, Miser J, Chatterji D and Poplack DG (1984) Cytosine arabinoside cerebrospinal fluid kinetics. *Clin Pharmacol Ther* **35**: 826–830

© 2001 Cancer Research Campaign

## References

[PDF_00497] Balm M., Hammack J. (1996). Leptomeningeal carcinomatosis. Presenting features and prognostic factors.. Arch Neurol.

[PDF_00499] Bleyer W. A., Dedrick R. L. (1977). Clinical pharmacology of intrathecal methotrexate. I. Pharmacokinetics in nontoxic patients after lumbar injection.. Cancer Treat Rep.

[PDF_00502] Boogerd W., Hart A. A., van der Sande J. J., Engelsman E. (1991). Meningeal carcinomatosis in breast cancer. Prognostic factors and influence of treatment.. Cancer.

[PDF_00504] Chamberlain M. C., Kormanik P. A. (1997). Prognostic significance of coexistent bulky metastatic central nervous system disease in patients with leptomeningeal metastases.. Arch Neurol.

[PDF_00507] Chamberlain M. C., Kormanik P. R. (1997). Carcinomatous meningitis secondary to breast cancer: predictors of response to combined modality therapy.. J Neurooncol.

[PDF_00510] Fizazi K., Asselain B., Vincent-Salomon A., Jouve M., Dieras V., Palangie T., Beuzeboc P., Dorval T., Pouillart P. (1996). Meningeal carcinomatosis in patients with breast carcinoma. Clinical features, prognostic factors, and results of a high-dose intrathecal methotrexate regimen.. Cancer.

[PDF_00513] Giannone L., Greco F. A., Hainsworth J. D. (1986). Combination intraventricular chemotherapy for meningeal neoplasia.. J Clin Oncol.

[PDF_00515] Glantz M. J., Cole B. F., Recht L., Akerley W., Mills P., Saris S., Hochberg F., Calabresi P., Egorin M. J. (1998). High-dose intravenous methotrexate for patients with nonleukemic leptomeningeal cancer: is intrathecal chemotherapy necessary?. J Clin Oncol.

[PDF_00519] Glantz M. J., Jaeckle K. A., Chamberlain M. C., Phuphanich S., Recht L., Swinnen L. J., Maria B., LaFollette S., Schumann G. B., Cole B. F. (1999). A randomized controlled trial comparing intrathecal sustained-release cytarabine (DepoCyt) to intrathecal methotrexate in patients with neoplastic meningitis from solid tumors.. Clin Cancer Res.

[PDF_00524] Graham F. L., Whitmore G. F. (1970). The effect of-beta-D-arabinofuranosylcytosine on growth, viability, and DNA synthesis of mouse L-cells.. Cancer Res.

[PDF_00527] Grant R., Naylor B., Greenberg H. S., Junck L. (1994). Clinical outcome in aggressively treated meningeal carcinomatosis.. Arch Neurol.

[PDF_00533] Grossman S. A., Finkelstein D. M., Ruckdeschel J. C., Trump D. L., Moynihan T., Ettinger D. S. (1993). Randomized prospective comparison of intraventricular methotrexate and thiotepa in patients with previously untreated neoplastic meningitis. Eastern Cooperative Oncology Group.. J Clin Oncol.

[PDF_00537] Hitchins R. N., Bell D. R., Woods R. L., Levi J. A. (1987). A prospective randomized trial of single-agent versus combination chemotherapy in meningeal carcinomatosis.. J Clin Oncol.

[PDF_00543] Jayson G. C., Howell A., Harris M., Morgenstern G., Chang J., Ryder W. D. (1994). Carcinomatous meningitis in patients with breast cancer. An aggressive disease variant.. Cancer.

[PDF_00546] Kim S., Chatelut E., Kim J. C., Howell S. B., Cates C., Kormanik P. A., Chamberlain M. C. (1993). Extended CSF cytarabine exposure following intrathecal administration of DTC 101.. J Clin Oncol.

[PDF_00553] Nakagawa H., Murasawa A., Kubo S., Nakajima S., Nakajima Y., Izumoto S., Hayakawa T. (1992). Diagnosis and treatment of patients with meningeal carcinomatosis.. J Neurooncol.

[PDF_00556] Ongerboer de Visser B. W., Somers R., Nooyen W. H., van Heerde P., Hart A. A., McVie J. G. (1983). Intraventricular methotrexate therapy of leptomeningeal metastasis from breast carcinoma.. Neurology.

[PDF_00559] Shapiro W. R., Young D. F., Mehta B. M. (1975). Methotrexate: distribution in cerebrospinal fluid after intravenous, ventricular and lumbar injections.. N Engl J Med.

[PDF_00562] Zimm S., Collins J. M., Miser J., Chatterji D., Poplack D. G. (1984). Cytosine arabinoside cerebrospinal fluid kinetics.. Clin Pharmacol Ther.

